# Collaborative writing: Strategies and activities for writing productively together

**DOI:** 10.1007/s40037-021-00668-7

**Published:** 2021-05-07

**Authors:** Lorelei Lingard

**Affiliations:** Department of Medicine, Centre for Education Research & Innovation, Schulich School of Medicine & Dentistry, Western University, London, ON Canada

In the Writer’s Craft section, we offer simple tips to improve your writing in one of three areas: Energy, Clarity and Persuasiveness. Each entry focuses on a key writing feature or strategy, illustrates how it commonly goes wrong, teaches the grammatical underpinnings necessary to understand it and offers suggestions to wield it effectively. We encourage readers to share comments on or suggestions for this section on Twitter, using the hashtag: #how’syourwriting?

Scientific writing is rarely a solo act. It’s not that the researcher doesn’t sit the same lonely vigil as the novelist, hunched over her laptop at the kitchen table in a winter dawn, hoping for inspiration. Sure she does. But, unlike the novelist, the research writer is rarely the sole architect of the text she’s creating. She is *sitting* alone at that table, but she is not *writing* alone. She writes on behalf of a team of collaborators, although she might wonder with the faintest tinge of resentment whether they are still in their warm beds as she sits in the pale morning light. Her sense of isolation is temporary though. It will dissipate at the precise moment when five email messages ping into her inbox, each one offering its unique feedback and edits on her circulated draft.

Writing collaboratively can be the best of times and the worst of times. At best, it is richly rewarding. Collaborators brainstorm the vision of the piece together; they enhance the story by thoughtfully questioning one another’s ideas; they craft the text iteratively, weaving a subtle tapestry of argument. At worst, it is deeply frustrating. Collaborators exchange ideas that don’t cohere; they compete to pull the story in pet directions that both complicate and dilute it; they manufacture a stitched-together, Frankenstein of a text. Leading a collaborative writing effort, therefore, is a tricky business. And while many resources exist to help structure and support collaborative research [1, 2], most pay little attention to the activity of collaborative writing, beyond issues of authorship candidacy.

Upcoming Writer’s Craft instalments will help you cultivate productive, satisfying writing relationships within your research team. In this piece, we make explicit the strategies and activities involved when a group of researchers writes together, so that your research team can identify them and discuss how they will unfold in a particular project.

## Strategies for collaborative writing

Collaborative writing is *“an iterative and social process that involves a team focused on a common objective that negotiates, coordinates, and communicates during the creation of a common document”* [3]. Collaborative writing can follow many different strategies [4], but five are most common [2]. These are one-for-all writing, each-in-sequence writing, all-in-parallel writing, all-in-reaction writing and multi-mode writing. Each offers a different approach to coordinating the work of writing in a group, and each is suited to different collaborative contexts.

“One-for-all writing” occurs when one person writes on behalf of the team. This strategy is appropriate when the writing task is simple and the stakes are low. For instance, many collaborative teams have a single author write an analytical memo describing the group’s discussion at a research meeting. One-for-all writing offers stylistic consistency and efficiency, but can limit consensus building or revision unless these are explicitly built into document cycles. Therefore, it is best used by groups with a shared understanding of the writing task. Alternately, it can serve as an efficient, low-stakes way of producing a first rough draft that the team understands will undergo multiple iterations using a range of other writing strategies. Writing a first draft is, of course, never ‘simple’, but when the agreed goal is ‘to get something on the page for us to work on together’, one-for-all writing can work well.

“Each-in-sequence” writing occurs when one person starts the writing, completes their task and passes it on to the next person to complete theirs. This strategy is useful for groups working asynchronously who cannot meet often and document-sharing platforms play a central role in its successful realization [5]. Many teams will use it in the early stages of drafting a grant application, for instance, because it allows for straightforward coordination of distributed work. The sequence may be purposeful: for example, the lead author will draft the introduction, then the research assistant will draft the methods, then a third team member will draft the results, at which point the piece will return to the lead author to draft the discussion. In practice, however, the sequence is often more random: writers get to their sections when they can. Each-in-sequence writing introduces a number of challenges, including minimal social interaction, one-person bottlenecks, lack of coherence because differing ideas are not reconciled or writers invalidate one another’s work, and haphazard version control. Together, these can result in poor overall coherence of the document. Teams can address these challenges by early meetings to clearly articulate the writing tasks and discuss areas of potential overlap or conflict. Also critical is agreement on the paper’s main story and how it will thread through all sections, as well as a shared approach to writing style basics such as first or third person narration, and active or passive voice construction. Coherence is also improved by assigning a lead writer who oversees the sequence and takes responsibility for integration. However, this writer must have the authority to successfully fulfill this role.

“All-in-parallel” writing involves dividing the writing work into discrete units and writers working simultaneously rather than in sequence. This strategy works well in situations where the writing task is easily divided and individual sections are not mutually dependent. Because it tends to offer more process efficiency and writer autonomy than each-in-sequence writing, all-in-parallel writing can produce rapid, high volume output. The strategy is most effective when divisions of labour are not arbitrary but planned according to each writer’s core expertise. For instance, the methodologist on a research team might write the first draft of the methods section, while a team member versed in the substantive domain of the work writes the literature review. The main challenge of all-in-parallel writing is that writers are blind to each other’s work while writing, which can produce redundant or contradictory material. To mitigate this, parallel writing requires careful pre-planning, including an outline of how the parts relate to one another, a shared vision of the audience and purpose of the document, and process to reconcile stylistic differences.

When researchers create a document together in real time, adjusting to each other’s changes and additions without explicit preplanning and coordination, they are using the strategy of “all-in-reaction” writing. Imagine, for example, that you write the first draft of a paper’s Problem/Gap/Hook and send it to your co-authors simultaneously for review and response. They may make edits simultaneously, their edits may contradict or concur with you or with one another, and they may be carefully considered or spontaneous and impulsive. An advantage of the all-in-reaction collaborative writing strategy is that it can support consensus through fluid and creative expression of all writers. It can also provoke debates and enable new, unexpected meanings to emerge. Its main disadvantages include limited coordination, the potential for chaotic development of the piece, and difficulties with version control due to simultaneity of writing. And, for more novice or less powerful writers on the team, it can produce a turbulent, threatening experience. Therefore, all-in-reaction writing works best in small, non-hierarchical groups where all members feel safe to express their opinions. When these conditions are met, it can be a powerful strategy for interdisciplinary groups to create new meanings beyond the borders of conventional disciplinary thinking.

Many research teams use a combination of these strategies over the course of a writing project, called “multi-mode writing”. For instance, a graduate student may produce the first draft of their research manuscript (one-for-all), which is then reviewed sequentially by team members, either as their calendars allow (each-in-random sequence) or in a preplanned order (each-in-purposeful-sequence). Revisions are then produced by the graduate student (one-for-all), and each team member reviews closely one section of the revision according to their expertise (all-in-parallel). The abstract may be written (often hours before the conference submission deadline) on Google Docs or by flurry of emails, with all team members simultaneously helping to whittle the word count and prioritize the key messages (all-in-reaction). Ensuring that all writers are capable users of the technologies supporting the collaborative process is critical.

These five strategies offer a framework for thinking critically about your own collaborative writing practices. Ask yourself these questions:

- What strategies does our team employ?
- Are our strategies purposeful, selected according to the nature of the team and the needs of the project, or are they accidental?
- Do we explicitly discuss how we will coordinate the work, or do we tacitly enact the same strategy each time?
- Are we using each strategy in ways that maximize its affordances and minimize its challenges?
- Are we using technology appropriately to support our collaborative activities?

Being purposeful and explicit about your collaborative writing strategy can help your team to maximize its unique affordances and minimize its challenges.

## Activities of collaborative writing

Collaborative writing involves more than just *writing*. Writing researchers have identified seven core activities: brainstorming, conceptualizing, outlining, drafting, reviewing, revising and editing [2].

In brainstorming, the writing group creates a list of potential ideas for the paper. Through conversation and text, they consider how to best represent the findings, what they might say about those findings in relation to the research question, what storylines would make for a compelling Discussion [6], and what conversations the piece might join in the literature. Brainstorming may start while data collection and analysis are still underway, particularly in qualitative research using theoretical sampling methods.

The activity of conceptualizing involves coalescing and prioritizing brainstorming ideas to articulate the central story of the paper. Some ideas will be set aside as insufficiently mature or irrelevant to the study’s main purpose; others will be pursued in ongoing analyses and reading of related theoretical and empirical literatures. When a study will yield more than one story, the process of conceptualizing must also consider the order and audiences of multiple manuscripts: which story should be told first? To whom?

Once the story is conceptualized, outlining is the process of detailing how it will unfold throughout the sections of the research manuscript genre. What needs to go in the introduction and what would be an unnecessary detour? What degree of detail should the methods include? Which results will be included and in what order? How will the discussion develop the ideas from the introduction? Outlining is an activity that can lend itself more readily to solo than to collaborative work. However, even if one writer takes the lead on outlining, the process should be visible to other members of the group. Talking through the outline in rough as a team, and then reviewing the outline created by the lead author, is one way to maximize both efficiency and input at this stage of the writing process.

In drafting, the outlined sections are flushed out into full sentences, paragraphs and arguments. Create a realistic schedule for this activity; an outline can seem like it lays the whole paper out, but the devil is in the details. Will the literature review be organized chronologically or by points of view in the current scholarly conversation? How much theoretical framing should appear in the introduction? How elaborate should the methods be, and what is the appropriate balance of description and justification? How will main results be illustrated, and which data should appear in tables, figures or quoted excerpts? How will the storyline develop in the discussion, beyond summary of results and limitations? In fact, when you acknowledge the complexity of the writing that goes into even a rough first draft, it probably makes more sense to draft sections in blocks. Consider pairing methods and results, and introduction and discussion, for instance, as these represent, respectively, the study and the story [7].

Reviewing, revising and editing usually occur in cycles. In reviewing, all members read draft material and provide feedback orally, by email, or in the text itself as track changes or comment boxes. Ideally, reviewing is a directed activity, in which members of the group are asked to focus on particular issues at specific points in the writing process. Revising involves the consideration, prioritization and integration of feedback from group members into the draft. Cycles of reviewing and revising will take place until the text is substantively complete, logically coherent, and rhetorically effective. Editing involves micro-level revisions for style, grammar and flow, which may take place either as individual sections mature or when the entire document is judged complete. Editing at this level may be an activity best undertaken by one writer on the team, in order that the paper does not read as though it was written by several individuals.

These collaborative writing activities are dynamic and iterative. Sometimes the storyline needs revisiting after a particularly substantive round of reviewing. Reviewing may shift into revising. Or editing may take place on some completed sections while other sections are still being reviewed. Because of this, successful collaboration requires cultivating a shared understanding of which activity is being undertaken at any given time. Are you finished brainstorming, you’ve agreed on a conceptualization and you’re now ready to outline the paper? If one writer thinks so, but another is still in brainstorming mode, this can impede progress. Are some writers providing review feedback at the level of micro-editing, while others are grappling with the conceptualization of the story as it is emerging in the draft? Is reviewing of a one-for-all draft turning into all-in-reaction revising? Having a language to talk about the different activities involved in collaborative writing can help to identify and resolve such disparate orientations to the work. And keep in mind that these activities are not ‘neutral’; they occur in the context of interpersonal dynamics on a research team. Collaborators mark, claim, defend and redraw intellectual territory as they work through the various activities associated with the writing [8]. Being attentive to enactment of territoriality throughout the writing process can help you focus on, rather than deflect, points of tension. Because within these may reside the team’s best opportunities to produce incisive, boundary-pushing thinking.

Depending on the writing project, these seven activities will receive variable emphasis and attention. Some results clearly dictate the storyline, making brainstorming less necessary. Some conceptualizations are sufficiently detailed that outlining can be more perfunctory. Some writers edit as they go, making the editing process less extensive at the end. The value of identifying these activities is to reflect on your own processes: does your writing team tend to skip some of these steps, such as outlining, and to what effect? Do some members of your writing team engage in some activities, such as reviewing, but not in others? Not every writer on a team will engage centrally in every activity. But some degree of participation in all of these writing activities yields more satisfying and efficient collaboration. For instance, team members not involved in the brainstorming and conceptualizing activities may inappropriately reintroduce through their reviewing and revising of drafts a storyline that the team had agreed to reserve for another paper. When such tensions in the writing emerge purposefully among collaborators engaged in all activities, they represent important moments for reviewing earlier decisions and perhaps reconceptualizing the piece. However, when they emerge incidentally because some collaborators are unaware of earlier activities, they can be a source of frustration and inefficiency.

## Conclusion

For your research collaboration to culminate in successful collaborative writing, you need to be able to break “writing” into its constituent activities and agree on strategies to coordinate them. This Writer’s Craft instalment offers a vocabulary to support you in this work.
